# Non-Human Primates as a Comprehensive Model for Studying Epigenetic Markers of Aging

**DOI:** 10.3390/genes17080905

**Published:** 2026-07-30

**Authors:** Viktoria M. Petrova, Evgeniia V. Simoroz, Natalia A. Dudko, Jelena Vasilevska

**Affiliations:** Department of Genetics, Research Center for Genetics and Life Sciences, Sirius University of Science and Technology, Sirius 354340, Russia; petrova.vm@talantiuspeh.ru (V.M.P.); simoroz.ev@talantiuspeh.ru (E.V.S.); dudko.na@talantiuspeh.ru (N.A.D.)

**Keywords:** non-human primates, epigenetics, aging, epigenetic clocks

## Abstract

Non-human primates (NHPs) serve as indispensable models for aging research due to their evolutionary proximity to humans, conserved epigenetic mechanisms, and lifespans amenable to longitudinal investigation. This review synthesizes age-related epigenetic modifications in NHPs, including DNA methylation, histone modifications, chromatin remodeling, and non-coding RNA regulation, and evaluates their alignment with human epigenetic markers. Epigenetic clocks developed on human data demonstrate robust predictive capacity in NHPs, and numerous age-associated methylation patterns are evolutionarily conserved. However, most epigenetic changes exhibit pronounced tissue specificity, with only a limited number of markers showing cross-tissue and cross-species consistency. Critical modulating factors such as sexual dimorphism, social hierarchy, environmental stressors, and early-life adversity significantly influence epigenetic aging trajectories. Current research remains restricted to a narrow subset of NHP taxa, predominantly macaques and baboons; expanding to include great apes would deepen our understanding of primate epigenetic aging. Advancing the field requires integrating multi-tissue, multi-species, and multi-omics approaches, including single-cell resolution analyses, to distinguish conserved mechanisms from lineage-specific adaptations. Such an integrative framework is essential for translating epigenetic discoveries into clinical interventions. By leveraging the unique advantages of NHPs, controlled interventional studies, longitudinal multi-tissue sampling, and causal mechanistic dissection, researchers can bridge basic discovery and therapeutic development. This approach promises to refine our understanding of aging biology and guide the rational design of next-generation therapeutics, from epigenetic modulators to lifestyle interventions, ultimately paving the way for personalized strategies that extend both lifespan and healthspan.

## 1. Introduction

Biological aging is characterized as a multifactorial process involving progressive physiological decline and increased susceptibility to age-related diseases; nevertheless, its precise risk factors and underlying molecular mechanisms remain largely unresolved. This functional deterioration is orchestrated by a set of canonical hallmarks encompassing both genetic and epigenetic alterations. Among these, aging-associated epigenetic modifications, including alterations in DNA methylation patterns, histone modifications, chromatin remodeling, non-coding RNA (ncRNA)-mediated regulation, and RNA modifications, can serve two distinct roles: they function both as biomarkers that track the passage of biological time and as mechanistic drivers that actively influence the aging trajectory and facilitate the emergence of age-related pathologies [1,2]. Notably, experimental interventions targeting epigenetic regulation have demonstrated efficacy in alleviating age-associated functional decline and extending lifespan across various animal models. Although rodents remain indispensable models for studying genetics and physiology, owing to their low cost, short lifespan, and genetic similarity to humans for many genes, the extrapolation of findings to human aging necessitates caution. Major limitations arise from fundamental interspecies differences in epigenetic clocks, biomarkers of aging, metabolic processes, and telomere biology [3]. While epigenetic clock models, which estimate biological age based on DNA methylation patterns, are applicable to both mice and humans, and while age-related methylation changes frequently target homologous genomic regions, direct translation of results is challenged by distinct rates and patterns of aging across species. Critically, the divergence between murine and primate aging extends to fundamental molecular pathways, rodents exhibit markedly different telomere dynamics, metabolic rates, and stress responses that limit their predictive validity for human aging interventions [4].

**Figure 1:**
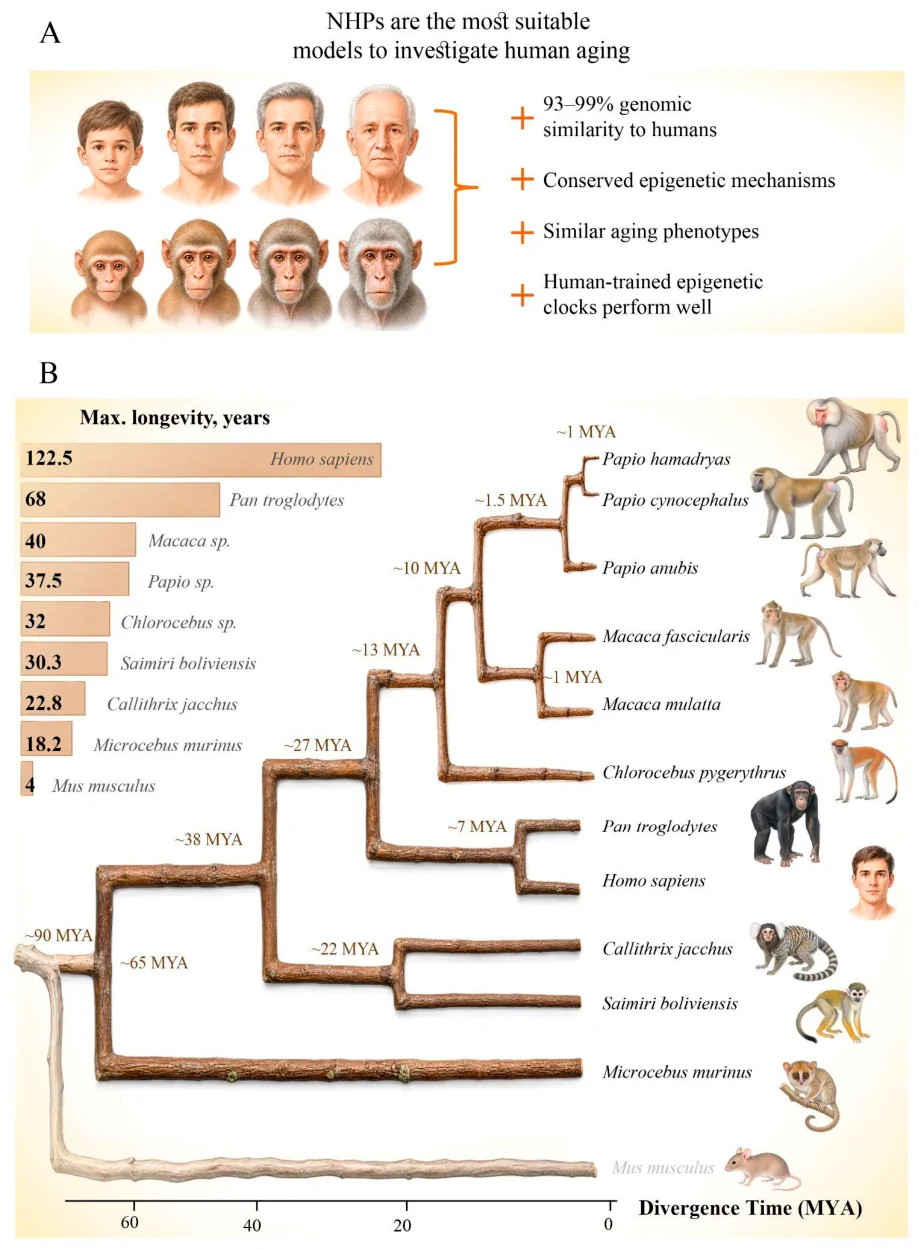
An overview establishing non-human primates as the premier model system for aging research due to their unique relevance to human biology. (**A**) Shared biological hallmarks of aging in humans and non-human primates. (**B**) Maximum longevity and phylogenetic tree comparing the most relevant non-human primates (NHPs) for aging studies with humans and mice. The tree was constructed using complete mitochondrial genomes with MEGA X v11.0.13 [15]. MYA, million years ago. Maximum longevity data were sourced from the AnAge Database [16] and reflect captive records. For genera represented by multiple species in the phylogenetic tree (*Papio*, *Macaca*), or for species lacking a direct AnAge entry (*Chlorocebus pygerythrus*), genus-level estimates were applied, derived from the maximum recorded longevity among congeneric species.

Non-human primates (NHPs) comprise all members of the primate order excluding humans and represent promising models for studying the aging process (Figure 1A). They are highly diverse and classified broadly into two main groups: prosimians (e.g., lemurs, lorises) and simians (monkeys and apes) [5]. Of particular relevance to aging research, NHPs offer several compelling advantages over rodent models. First, their close evolutionary proximity to humans (93–99% genomic similarity) extends to conserve epigenetic mechanisms such as DNA methylation and chromatin remodeling [6,7]. Second, NHPs exhibit human-like aging phenotypes, including immune senescence, cognitive decline, and metabolic changes, within lifespans amenable to longitudinal studies. Third, epigenetic clocks trained on humans perform well in NHPs, with age-related methylation changes occurring at conserved CpG sites [8,9]. Additionally, controlled research settings in NHPs minimize confounding variables while enabling ethical interventions (e.g., caloric restriction, pharmaceuticals) not feasible in humans.

However, three observations temper this characterization. First, primates do not constitute the longest-lived mammalian order, whether measured by absolute lifespan, longevity adjusted for body size, or metabolic expenditure over a lifetime. Second, although relative brain size correlates strongly with longevity in primates, this relationship represents an aberrant trend among mammals generally, and other organ systems account for an even greater proportion of lifespan variation. Third, no progressive evolutionary increase in longevity has occurred across primate superfamilies. Upon weighing the strengths and limitations of NHPs as model systems for epigenetic aging research, it becomes evident that their principal value does not reside in the faithful recapitulation of human aging phenotypes per se, but rather in their capacity to elucidate evolutionarily conserved epigenetic mechanisms. This distinctive attribute renders NHPs particularly valuable from a translational and comparative perspective.

In keeping with evolutionary senescence theory, the remarkable longevity observed in primates may instead be attributable to an evolutionary history characterized by low vulnerability to environmentally imposed mortality, a circumstance shaped by their body size, arboreal habits, and propensity for social grouping [10]. As comprehensively outlined in a recent review [11], a set of NHP species has been identified as particularly suitable models for aging research, a selection that is substantiated by their phylogenetic proximity to humans, in marked contrast to phylogenetically distant species such as mice (Figure 1B). Among these primates, different species offer complementary advantages for investigating the aging process and its epigenetic regulators. The common marmoset, for example, is especially advantageous due to its modest body size, comparatively short generation time, and high fecundity, which collectively facilitate longitudinal tracking and experimental interventions. In parallel, macaques and chimpanzees remain preferred models owing to their extensive physiological and genetic homology with humans, alongside their long-standing and thoroughly validated use in biomedical research.

NHPs provide distinct experimental advantages beyond their phylogenetic closeness to humans, advantages that are unattainable in human research. They allow for the collection of biomaterials from multiple tissues, including brain regions, vascular tissues, and various organs, across different developmental and aging stages, which cannot be ethically obtained from living human subjects [12,13]. The examination of post-mortem brain tissues from longitudinally studied NHP cohorts facilitates direct correlations between behavioral trajectories and neuropathological changes. This approach is limited in human studies due to tissue collection occurring only post-mortem, often after prolonged postmortem intervals. Longitudinal sampling throughout the lifespan, combined with controlled environmental and lifestyle interventions, enables the investigation of epigenetic aging dynamics that are not feasible in human cohort studies. Species such as marmosets and macaques can be maintained under strictly regulated conditions, including photoperiod, temperature, and diets allowing for rigorous control of confounding variables that typically affect human observational research. Interventional studies involving caloric restriction, pharmacological agents (e.g., rapamycin, metformin), and environmental modifications can be precisely monitored in NHPs, thereby providing causal insights into the mechanisms of epigenetic aging that human studies cannot establish [12,13,14]. These causal intervention studies, coupled with the capacity to collect brain and other tissues from animals of known ages under controlled conditions, underscore the indispensable role of NHPs as translational models for elucidating the mechanisms underlying human aging.

Given these considerations, this review aims to achieve several objectives: to demonstrate that NHPs share the same molecular and epigenetic pathways as humans during aging, establishing their validity as translational models. Next, to critically evaluate whether NHPs exhibit additional or distinct age-associated epigenetic features beyond those observed in humans, thereby informing the appropriate interpretation of NHP-derived data for human aging. Additionally, to identify the specific contribution that NHP studies have made in investigating tissue-specific aging mechanisms, particularly the brain, thereby revealing aspects of epigenetic aging that cannot be studied through human biomaterial alone. By systematically comparing age-associated epigenetic markers in NHPs against those well documented in human aging, this review evaluates the suitability of NHPs for translational epigenetic research and identifies critical gaps that remain to be addressed.

## 2. Materials and Methods

This narrative review was conducted based on a systematic literature search across multiple databases, including PubMed, Web of Science, Scopus, and Google Scholar. The search encompassed publications from January 2000 through December 2025, with the final update performed in June 2026. The search strategy incorporated keywords pertaining to aging, epigenetic mechanisms, non-human primate species, epigenetic clocks, and pharmacological or dietary modulators. The review primarily included original research articles; review articles were utilized exclusively to identify primary sources and provide contextual background. Both peer-reviewed publications and preprints (e.g., from bioRxiv) were considered when they presented novel data not yet available in the peer-reviewed literature. Given the narrative nature of this review, a formal meta-analysis was not undertaken. Instead, findings were synthesized qualitatively through several approaches: (1) thematic categorization, grouping studies by epigenetic mechanisms such as DNA methylation, histone modifications, and non-coding RNA; (2) cross-species comparison, evaluating markers and epigenetic clock performance across non-human primate species and in relation to human data; (3) tissue-specific analysis, organizing findings by tissue type to distinguish conserved from tissue-specific markers; and (4) modulator analysis, separately examining studies addressing sex differences, social factors, stress, and interventions to identify consistent patterns. No novel data were generated or analyzed in this study; all data presented are derived from previously published sources, which are duly cited in the reference list.

## 3. DNA Methylation Changes

Key epigenetic shifts in aging involve DNA methylation changes that modulate gene expression. A hallmark of this process is age-related global hypomethylation, which contributes to genomic instability, heightened expression of inflammatory markers and oncogenes, and the reactivation of transposable elements. Meanwhile, specific CpG sites within the genome may either gain or lose methyl groups over time, and their positioning within gene regulatory regions critically determines the resulting effects on transcription [17]. Gene promoters characterized by high CpG density are notably prevalent in long-lived species, such as primates and other mammals. This genomic feature is thought to represent an adaptive protective mechanism that safeguards regulatory stability over an extended lifespan [18]. In a study examining universal mechanisms of age-related DNA methylation across 185 mammalian species, including NHPs and humans, several recurring age-associated markers were identified. Hypermethylation was observed at CpG sites located within Polycomb repressive complex 2 (PRC2) binding regions and bivalent promoters that govern developmental genes. Conversely, hypomethylation occurred at promoters linked to circadian rhythm and mitochondrial function, both of which exhibit age-related decline and dysregulation. Collectively, these findings indicate that age-associated methylation patterns are evolutionarily conserved across mammals [19].

### 3.1. Age-Associated DNA Methylation Markers

A clear differentiation must be established between epigenetic markers of aging, specifically, measurable changes in CpG methylation that correlate with chronological or biological age, and the molecular epigenetic mechanisms that actively drive the aging process. While such markers serve as valuable biomarkers for estimating age and predicting disease risk, they do not necessarily indicate causality. Conversely, epigenetic factors refer to the mechanistic modifications in gene expression and chromatin configuration that directly contribute to functional decline. This section primarily concentrates on the identification and characterization of methylation markers validated across NHP species, whereas the subsequent section addresses the functional epigenetic mechanisms that regulate the aging process.

In a comprehensive 2023 study, Horvath and colleagues profiled DNA methylation across 2400 tissue specimens from 37 primate species. Their results reveal that certain epigenetic aging signatures are deeply conserved throughout the primate order. Hypermethylation with age was prominently detected at loci such as *KLF14*, *LHFPL4*, *LHFPL3*, *BDNF*, *TBR1*, *FOXG1*, and several *HOX* genes (e.g., *HOXC4*). Notably, the overrepresentation of *HOX* family members and PRC2 targets points to a mechanistic link between developmental regulation and the epigenetic clock. In contrast, hypomethylation emerged in genes including *TRPS1*, *SNX1*, *SMG6*, *ARID5B*, and *EWSR1* [20]. The study further confirmed that the majority of methylation changes tied to age are tissue-dependent, an observation subsequently reinforced by more focused primate studies. For instance, research on rhesus macaques (aged 1.8 to 42 years) identified tissue-specific age-associated markers across multiple organs, with notable examples including *CHD3* in adipose, *VGF* in blood, *PAX6* in cortex, *AGAP3* in kidney, *ONECUT2* in liver, *GRIA1* in lung, *MN1* in muscle, and *LHFPL4* in skin. Despite this tissue specificity, the *KLF14* gene stood out as a cross-tissue candidate: four CpG sites in its promoter exhibited age-related hypermethylation in fat, blood, cerebral cortex, and skin. Additionally, age-related hypomethylation was observed at binding motifs for transcription factors *ZIC1/ZIC2* in muscle and *TFAP2C* in blood and lungs, suggesting that such epigenetic changes may disrupt transcription factor binding and thereby alter the regulation of downstream target genes during aging [21]. A follow-up macaque study added another layer: age-related methylation changes tend to cluster in regions that define a tissue’s unique epigenetic identity. On average, about 12% of tissue-specific markers overlapped with aging markers—but this varied hugely, from less than 5% in blood to over 40% in the thymus. Furthermore, the effect of aging on tissue identity was bidirectional: in blood and heart, it eroded tissue-specific methylation (loss of identity), whereas in thymus and pituitary, it enhanced it (gain of identity). Nevertheless, the authors stress that tissue-specific markers overwhelmingly dominate the epigenetic landscape [22]. Zooming in on immune cells from macaque blood, another study examined age-related methylation changes at the level of transcription factor binding motifs [23]. Hypomethylated DMRs were enriched for motifs recognized by factors involved in oxidative stress responses (*Bach1*) and age-related inflammation (*NFE2L2* and components of the AP-1 complex). In contrast, hypermethylated DMRs were enriched for motifs of ZBTB family transcription factors, known developmental regulators, as well as *NRF1*, which activates metabolic gene expression. Notably, hypermethylation also targeted motifs for *ZBTB33* (KAISO), a transcriptional regulator associated with histone deacetylation and heterochromatin formation, processes that suppress inflammatory gene expression, proliferation, and apoptosis. Collectively, these immune-cell-specific methylation patterns point to a dual-age-related shift: activation of inflammatory responses and oxidative stress, coupled with suppression of metabolic pathways.

Another primate model, the vervet monkey, which is evolutionarily close to the rhesus macaque, was investigated in a study that assessed aging-associated DNA methylation markers across three tissue types: blood, liver, and cerebral cortex from animals ranging from 0 to 25 years of age. Intertissue correlations for methylation changes were moderate between blood and liver (R  = 0.63), but notably low between blood and cerebral cortex (R  = 0.14), as well as between liver and cerebral cortex (R  = 0.21). The authors identified tissue-specific hypermethylated markers, including the *KIAA0408* exon and SST promoter in blood, the *KCNC4* exon and *HOXC4* promoter in the cerebral cortex, and the *FOXG1* promoter and *SIM1* intron in the liver. Hypomethylated markers were also detected, such as the *Zfp161* motif in the cerebral cortex and immune-related transcription factor motifs (*Jundm2*, *FOS*, *JUN*, and *CREB*) in the liver. Across all three tissues, common age-associated markers were observed: hypermethylation accumulated at exons of *LHFPL4* and *FOXD3*, as well as the *TLX3* promoter, while loss of methylation occurred at the *SP1* exon, a CpG site downstream of *CD46*, and the *TRPS1* intron [24]. As seen in other mammals, including primates [19,20], age-related hypermethylated CpGs in vervet monkeys were consistently found in all tissues near target genes of the Polycomb proteins *SUZ12* and *EED*. Additionally, loss of methylation at the *TFAP2C* transcription factor binding motif was observed universally across tissues.

Marmosets belong to a distinct evolutionary branch, the New World monkeys. Their comparatively short lifespan, relative to macaques or vervet monkeys, makes them an advantageous model for long-term longitudinal studies. An analysis of DNA methylation markers in marmoset blood showed that the majority of age-associated CpG sites located in promoter regions and 5′-untranslated regions of the genome tend to become hypermethylated with age. Among these hypermethylated markers, the most notable changes were observed at the *ANK1* exon, the *SCG3* promoter, and the *UNC79* exon. Regarding transcription factor binding motifs, only *NFYA*, which has been implicated in the pathogenesis of cancer and rheumatoid arthritis, exhibited age-related alterations [25].

As hominid primates and close genetic relatives of humans, chimpanzees have served as valuable models for studying DNA methylation in the aging process. In a 2012 study by Garagnani and colleagues, pyrosequencing was used to assess three target genes: *ELOVL2*, *CCDC102B*, and *ZNF423*, in animals ranging from 2 to 39 years of age. Among these, only *ELOVL2* showed a statistically significant association with advancing age [26]. This mirrors earlier findings in humans, where the same gene is known to acquire increasing methylation marks over time, reinforcing its potential as a conserved biomarker of biological aging across species [27]. In contrast, no age-related changes were observed for the other two genes examined.

Beyond gene-specific methylation changes, the age-related activation of mobile genetic elements has also emerged as a marker of biological aging, with evidence reported in both humans [28] and non-human primates, such as crab-eating macaques [29]. Supporting this, a 2024 study by Watowich and colleagues found a significant association between CpG sites within transposons and aging in rhesus macaques. Specifically, older individuals exhibited reduced transposable element (TE) methylation, a shift that may facilitate TE reactivation. Such reactivation is thought to promote genomic instability, contribute to the accumulation of double-stranded DNA breaks, and trigger inflammatory responses, hallmarks of the aging process [23]. Age-related activation of transposons, caused by their epigenetic dysregulation, is a well-known evolutionarily conserved mechanism of aging, observed in various mammals, including humans [30,31].

**Figure 2:**
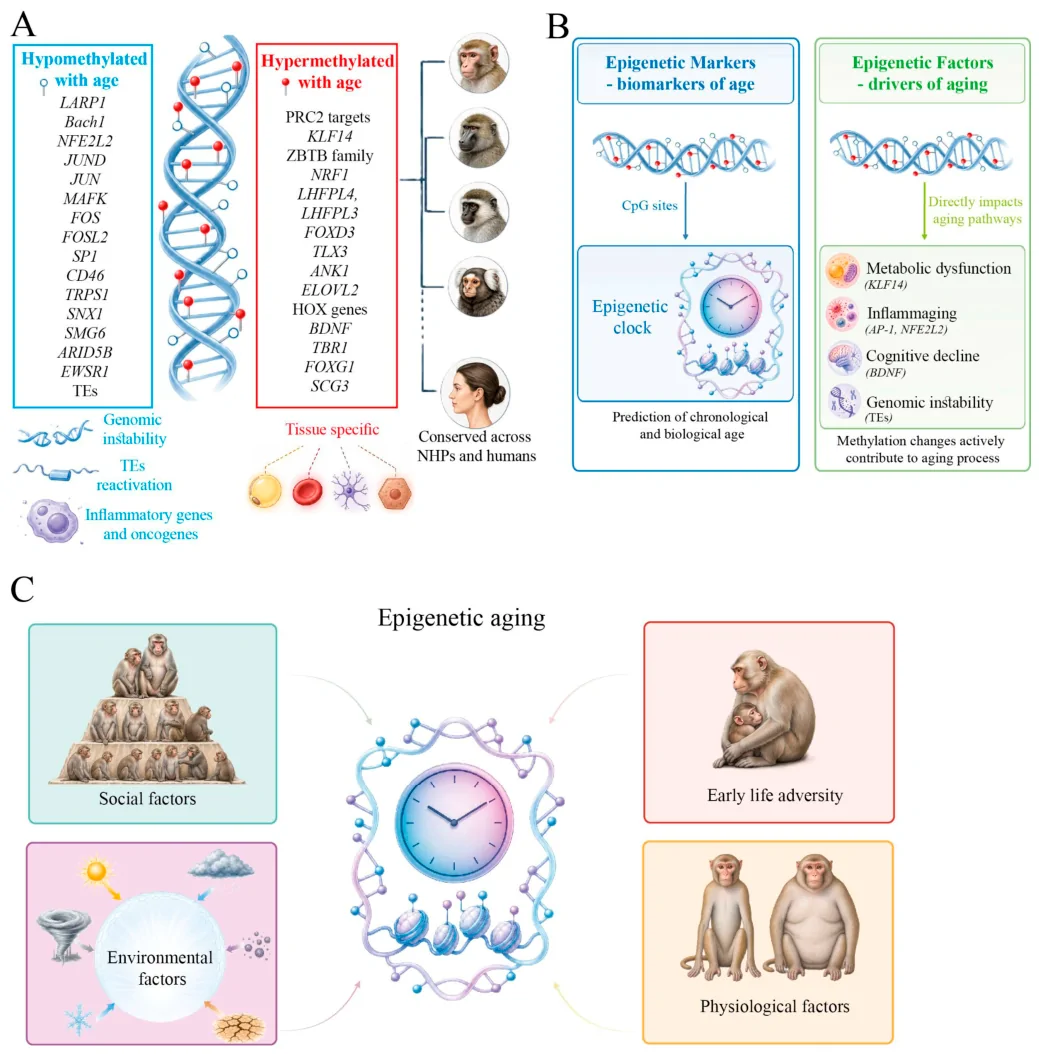
The landscape of DNA methylation changes in primate aging. (**A**) A summary of evolutionarily conserved age-associated DNA methylation markers identified across NHPs with parallel findings confirming their involvement in human aging. Genomic hypomethylation (blue) is mostly associated with genomic instability and transposon reactivation, whereas hypermethylation (red) is often tissue-specific. (**B**) The conceptual distinction between CpG sites used as epigenetic clock biomarkers for age prediction and those in genes that act as functional drivers of the aging process. (**C**) Key modulating factors that shape epigenetic aging trajectories in primates.

In summary, the aging process in primates is consistently accompanied by DNA methylation changes that are largely conserved with humans (Figure 2A). However, these age-related epigenetic alterations are not uniform across the organism; rather, they exhibit marked tissue specificity, with certain tissues accumulating methylation shifts at accelerated rates compared to others. This differential dynamics underscores the complexity of epigenetic aging and suggests that tissue context must be carefully considered when interpreting methylation-based biomarkers or extrapolating findings across species.

### 3.2. Epigenetic Clocks as Markers of Biological Age

The identification of CpG sites that display a strong correlation between methylation alterations and chronological age has facilitated the development of mathematical models designed to estimate the pace of epigenetic age-related changes, commonly termed epigenetic clocks. It is important to recognize that epigenetic clocks are markers of biological aging, they provide a statistical estimate of age based on methylation patterns, but the specific CpGs they incorporate may not be causal factors in the aging process. Rather, they serve as composite biomarkers that integrate the cumulative effects of aging across the epigenome.

The first epigenetic clock capable of predicting human chronological age, introduced by Gregory Hannum in 2013, incorporated 71 CpGs but was restricted only to peripheral blood samples [32]. In the same year, Steve Horvath unveiled a more versatile clock featuring 353 CpGs that performed reliably across a broad spectrum of human tissues [33]. Nevertheless, chronological age often fails to capture the true physiological condition of tissues, as external factors can either accelerate or decelerate the aging process [34]. To address this issue, second-generation epigenetic clocks were subsequently developed, with a focus on evaluating clinical health markers and forecasting the risk of mortality from all causes [35,36]. Building on this progress, a third-generation clock has emerged, specifically intended to quantify the pace of tissue aging [37].

Given the remarkable evolutionary conservation of mammalian aging mechanisms, subsequent efforts led to the creation of pan-mammalian epigenetic clocks that demonstrate robust predictive performance (R > 0.96) across 59 tissue types from 185 mammalian species, including diverse primate representatives [19]. Based on these results, the HorvathMammalMethylChip40 microarray was produced, which later widely was used to build customized epigenetic clocks for individual mammalian species, in particular NHPs [20,21,24,25].

Notably, the close phylogenetic relationship between primates and humans is reflected in the high transferability of primate-derived epigenetic clocks to human applications. Applying a comprehensive dataset from 11 harporhine species and 26 strepsirrhine species, Horvath et al. (2023) developed two highly accurate epigenetic clock models, one for chronological age estimation (R = 0.99), and another for relative age prediction (R = 0.96). Importantly, the chronological age model retained exceptional precision when applied to human samples (R = 0.98), underscoring the strong cross-species concordance and underscoring the potential of primate epigenetic clocks as direct translational proxies for human biological age assessment [20]. However, even with a universal pan-primate clock, the specific primate type remains an important factor (Table 1).

#### 3.2.1. Vervet Monkey (*Chlorocebus sabaeus*)

In 2021, a research team led by S. Horvath developed an epigenetic clock using vervet monkey models, constructing separate clocks for three distinct tissue types: peripheral blood, liver, and the prefrontal cortex (Brodmann area 10). Separate clock models were developed for each tissue individually, alongside a pan-tissue clock and a bispecies vervet–human clock. The sustained predictive accuracy of these vervet–human hybrid models provides compelling evidence for the evolutionary conservation of epigenetic aging signatures across primates. When these vervet-derived clocks were applied to tissue samples from a phylogenetically more distant primate, the rhesus macaque, the models yielded high age correlations albeit with notable systematic offsets, suggesting shared epigenetic aging dynamics modulated by species-specific intercepts. The pan-tissue vervet clock, when tested for cross-species age estimation in macaques and humans, demonstrated moderate predictive performance (R = 0.76 in rhesus macaques and R = 0.64 in humans), indicating that while some degree of age-related epigenetic information is conserved, tissue- and species-specific calibration remains essential for precise age prediction across primate taxa [24].

#### 3.2.2. Common Marmoset (*Callithrix jacchus*)

Researchers constructed chronological and relative age (chronological/maximum lifespan) predictors using blood samples from the common marmoset. The implementation of relative age clocks facilitates comparative analyses across species by normalizing for differences in longevity. For example, a relative age clock constructed for both marmosets and humans exhibited high predictive accuracy when applied jointly to samples from both species (correlation coefficient R = 0.96), although its performance was comparatively reduced when applied exclusively to marmoset samples (R = 0.86). These findings suggest that the biological mechanisms underlying aging are highly conserved across evolutionarily distant taxa and underscore the utility of marmosets as a model organism for evaluating geroprotective interventions. Furthermore, species-specific chronological age clocks developed for marmoset blood demonstrated robust accuracy (R = 0.95) with a median absolute error (MAE) of 0.72 years. Nevertheless, cross-species validation indicated limited transferability: while the marmoset-based model maintained reasonable accuracy when applied to vervet monkeys (R = 0.83), its predictive capacity was markedly diminished in rhesus macaques (R = 0.30). Importantly, the application of a cross-species relative age model significantly enhanced predictive performance in macaques (R = 0.79) [25].

#### 3.2.3. Rhesus Macaque (*Macaca mulatta*)

The rhesus macaque serves as the primary primate model in aging research. In a study by S. Horvath et al., a macaque-specific epigenetic clock was constructed using eight tissue types, predominantly skin (51) and blood (199). Several clock variants were generated: pan-tissue (R = 0.95, MAE = 1.4 years), skin-specific, blood-specific, and a macaque–human clock designed to measure both chronological age (R = 0.98 for combined species, R = 0.95 for macaques alone) and relative age (R = 0.97 for combined, R = 0.95 for macaques alone). The pan-tissue macaque clock demonstrated strong cross-species age correlation in another Old World primate, the vervet monkey, with R values of 0.96 in blood, 0.92 in cortex, and 0.98 in liver. However, prediction accuracy varied by tissue, with median errors of 9 years for cortex, 1.9 years for blood and 3.7 years for liver. A macaque–human model also yielded a relatively high correlation for vervet age prediction (R = 0.89) [21].

In a separate study [39], the authors applied Reduced Representation Bisulfite Sequencing (RRBS), which enables interrogation of a greater number of CpG sites than the HorvathMammalMethylChip40 microarray used in the study by S. Horvath et al. [21]. Two modeling approaches were tested: a classical site-based model, which achieved a median absolute deviation (MAD**^2^**) of 2.11 years, and a window-based model, which averaged methylation values over small genomic regions and proved more accurate (MAD**^2^** = 1.42 years). The resulting RheMacAge model comprised 359 windows, of which 164 exhibited age-related hypomethylation and 195 showed hypermethylation. RheMacAge performed well on an independent macaque cohort (R = 0.69, MAD**^2^** = 2.09 years), confirming its generalizability across populations. When applied to baboons, the model also yielded promising results. Although RheMacAge underperformed relative to a specialized baboon clock (MAD**^2^** = 0.85 years for males, 1.6 years for females), it captured the same underlying biological signal, including confirmation of an association between high social status and accelerated epigenetic aging in males (R = −0.47) [39]. The authors further tested their clock on data from macaques affected by Hurricane Maria [23] and found no association with epigenetic age alterations.

A tissue-specific epigenetic clock developed in rhesus macaques demonstrated high accuracy in predicting chronological age, with MAE values ranging from 0.82 to 1.53 years depending on the tissue and correlations reaching R = 0.95. Individual deviations in age prediction were consistently observed across most tissues, with individual identity accounting for 19% of the variance in age predictors (after controlling for sex, age, group, and tissue type). This suggests that the epigenetic clock tends to run consistently faster or slower across the majority of tissues, indicating that aging is a coordinated, organism-level process. No distinct subgroups of individuals with tissue-specific aging patterns were identified, implying that age-related heterogeneity is largely generalized. Moreover, intra-individual heterogeneity in predicted age increased with chronological age, pointing to growing intertissue epigenetic divergence over time [22].

#### 3.2.4. Baboon (*Papio cynocephalus/Papio anubis*)

The first epigenetic clock for estimating chronological age in Amboseli baboons was developed by Anderson et al. (2021) and comprised 573 CpG sites. It demonstrated high predictive accuracy, with a correlation of R = 0.762 and a median absolute difference (MAD**^3^**) of 1.1 years. Performance varied by sex: the clock achieved a MAD**^3^** of 0.85 years (R = 0.86) in males, compared to 1.6 years (R = 0.78) in females [41]. In a separate study using olive–yellow baboon hybrids, the authors constructed several epigenetic clock variants: a pan-tissue clock (R = 0.96, MAD**^2^** = 1.1 years), a brain-based clock incorporating two regions: cerebellum and cortex (R = 0.96), and a cortex-specific clock (R = 0.97). Building on these data, a human–baboon interspecies clock was also developed. For chronological age, accuracy reached R = 0.99 on combined human and baboon data and R = 0.94 on baboon tissue alone; for relative age, corresponding values were R = 0.97 and R = 0.95, respectively. Correlations of epigenetic age acceleration between different baboon tissues were weak, ranging from a maximum of R = 0.44 (cortex vs. cerebellum) to a minimum of R = 0 (heart vs. muscle, and fat vs. cerebellum). When tested on 16 human tissues (n = 1352), the pan-tissue baboon clock yielded moderate to high correlations depending on the tissue: skin (R = 0.93), heart (R = 0.76), kidney (R = 0.69), blood (R = 0.63), bone marrow (R = 0.33), and fat (R = 0.49). However, age calibration was poor due to high absolute errors, rendering this clock suitable only for estimating relative rather than precise chronological age in humans [20].

In another study, a separate model was constructed to predict chronological age in captive olive baboons using 153 CpG sites. Approximately 27% of the baboon sample exhibited accelerated aging (epigenetic age ≥ chronological age), roughly 28% showed decelerated aging (epigenetic age < chronological age), and the remaining individuals displayed age estimates consistent with their chronological age. Additionally, the baboon clock was subsequently applied to assess the relationship between accelerated epigenetic aging and two physiological measures: walking speed and fine motor skills. While older baboons exhibited slower walking speed and poorer fine motor performance, delta age (ΔAge), defined as the difference between epigenetic and chronological age, did not reliably predict either measure. This finding suggests that these two aging-related phenotypes are independent in this baboon cohort [42].

#### 3.2.5. Chimpanzee (*Pan troglodytes*)

The development of an epigenetic clock in chimpanzees, the closest evolutionary relative of humans, provides an opportunity to explore the balance between conservative and divergent epigenetic changes. Ito and colleagues developed an age-predictive model for chimpanzees by targeting the human-orthologous genes *ELOVL2*, *CCDC102B*, and *ZNF423*. Their analysis identified five informative CpG sites across these loci—four situated within *ELOVL2* and one within *CCDC102B*. The resulting epigenetic clock demonstrated a moderate correlation with chronological age (R = 0.741), but relatively large mean absolute deviation (MAD**^1^** = 5.42 years). Despite this, the directionality of age-related methylation changes mirrored human patterns: hypermethylation at *ELOVL2* and hypomethylation at *CCDC102B*, suggesting evolutionary conservation in the epigenetic regulation of aging between the two species [26].

In a larger study, Guevara et al. (2020) constructed a chimpanzee-specific epigenetic clock capable of predicting chronological age with a MAD**^2^** of only 2.4 years from known age, a level of precision comparable to that achieved by human clocks. Intriguingly, however, this chimpanzee clock exhibited limited overlap with previously established human epigenetic clocks, suggesting that despite the close evolutionary proximity between the two species, the specific CpG sites driving age-associated methylation changes are only partially shared [38]. To estimate age in chimpanzees, this study additionally applied the Hannum [32] and Horvath [33] epigenetic clocks which were originally developed for humans. The Hannum clock consistently overestimated chimpanzee age, yielding a MAD**^2^** of 5.2 years. In contrast, the Horvath clock produced considerably more accurate predictions, with a MAD**^2^** of 2.8 years. This improved performance is likely attributable to the fact that the Horvath model was trained on a diverse set of tissue types and may incorporate a higher proportion of conserved CpG sites that reliably track age-related changes across both humans and chimpanzees.

Given that aging is frequently linked to cognitive decline and the emergence of age-related neurodegenerative conditions, including Alzheimer’s and Parkinson’s diseases, epigenetic modifications in the brain have drawn considerable scientific attention. In this work [43], the authors employed both the chimpanzee epigenetic clock [38], which they had previously developed, and the standard Horvath clock [33] to evaluate epigenetic age across different brain regions in humans and chimpanzees. Their results indicated that, with advancing age, the brain undergoes epigenetic alterations at a slower pace than blood. Furthermore, in humans, the dorsolateral prefrontal cortex displayed more substantial epigenetic changes relative to the cerebellum, a finding that may be attributed to either the region’s extended maturation period or its heightened vulnerability to neurodegenerative processes. Notably, this regional disparity in aging rates between the dorsolateral prefrontal cortex and cerebellum was absent in chimpanzees.

Collectively, these results indicate that while substantial conservation underpins the fundamental mechanisms of epigenetic aging in hominids, there exists appreciable divergence in both the rate of age-related methylation change and its genomic distribution.

### 3.3. Age-Associated Methylation Changes in Genes Essential to the Aging Process

Certain methylation alterations extend beyond their role as biomarkers, occurring within genes whose encoded products are integral to the aging process. These modifications constitute molecular epigenetic factors of aging, as opposed to mere markers, due to their direct influence on cellular senescence, metabolic regulation, and longevity pathways. A prominent example is *KLF14*, which demonstrates age-associated hypermethylation across various tissues in rhesus macaques [21]. *KLF14* encodes a transcription factor involved in regulating adipogenesis, glucose metabolism, and insulin sensitivity; its epigenetic silencing with age likely contributes to metabolic deterioration. Similarly, hypermethylation of *BDNF*, which encodes brain-derived neurotrophic factors essential for neuronal survival and synaptic plasticity, represents a mechanistic contributor to cognitive decline and increased risk of neurodegenerative diseases. Age-related hypermethylation of *TBR1*, a critical regulator of cortical development and neuronal differentiation, may underlie impairments in neural function observed during aging. Furthermore, the consistent hypermethylation of *HOX* gene family members across primate species suggests that epigenetic dysregulation of developmental pathways is a fundamental aspect of aging, potentially impacting tissue renewal and regenerative capacity [20].

Conversely, hypomethylation at binding motifs for *Bach1* and *NFE2L2* functions as an epigenetic factor that derepresses oxidative stress response pathways. *NFE2L2* (also known as NRF2) orchestrates the antioxidant response, and its epigenetic dysregulation may result in compromised redox homeostasis with advancing age. Additionally, hypomethylation of components of the AP-1 transcription factor complex (*JUN, FOS, JUND*) indicates functional activation of inflammatory transcriptional programs, thereby linking epigenetic modifications directly to inflammaging [23]. The hypomethylation of transposable elements arguably represents the most direct functional epigenetic factor, as reactivation of these elements can induce genomic instability, activate innate immune responses via the cGAS-STING pathway, and actively promote cellular senescence [23,44].

The distinction between epigenetic markers and factors becomes particularly salient when considering that many CpG sites employed in epigenetic clocks, although highly predictive of chronological age, are not situated within genes with established roles in aging (Figure 2B). In contrast, methylation changes in functionally validated aging-related genes such as *KLF14*, *BDNF*, and *NFE2L2* possess strong mechanistic plausibility as drivers of age-associated decline. This differentiation holds significant implications for translational research: while epigenetic clocks serve as robust biomarkers for assessing biological age, elucidating the functional consequences of methylation alterations in key aging genes is imperative for the development of targeted interventions aimed at modulating the aging process.

### 3.4. Key Factors Shaping Epigenetic Aging

Although epigenetic clocks developed in primates have demonstrated remarkable precision as a universal primate aging marker and show promising correlations likely relevant to human aging, several important factors must be taken into account when interpreting epigenetic aging results in primates. For instance, studies on wild baboons, primates characterized by complex social structures and strict hierarchical group living, reveal that ongoing social dynamics significantly influence the aging process. In male baboons, both high social rank and increased body mass index were positively associated with epigenetic clock acceleration, with alpha males exhibiting an average age acceleration of 10.95 months relative to their chronological age. Researchers have proposed that this acceleration stems from the physiological burden of status maintenance and competitive aggression [41]. This pattern aligns with the well-documented shorter lifespan of high-ranking individuals, which is accompanied by elevated testosterone and glucocorticoid levels [45]. However, this parallel is not entirely straightforward for humans. Although in humans, social and environmental factors also play a substantial role in shaping the epigenetic clock, a high testosterone-to-estradiol ratio has been associated with a slowing of the epigenetic clock, an effect linked to its regenerative function through decreased methylation of the *PAI1* gene [46]. In contrast, a study on rhesus macaques reported no association between epigenetic aging rate and dominance rank in either sex. This discrepancy may stem from interspecific differences in rank acquisition mechanisms. In baboons, attaining and maintaining high rank entails substantial energetic costs and physical competition, accompanied by chronic stress. Rhesus macaques, by contrast, exhibit a less competitive social structure, where rank rises primarily with group tenure rather than through active contest [39].

Beyond social hierarchy and hormonal factors, stress itself emerges as a powerful modulator of epigenetic aging. This is particularly evident in the context of acute environmental stressors. For example, a study of 101 free-living rhesus macaques on Culebra Island, Puerto Rico, exposed to the devastating effects of Hurricane Maria, revealed widespread epigenetic alterations: methylation was changed at 32,048 CpG sites, some of which overlapped with age-associated sites in regions critical for active gene regulation, including promoters, enhancers, and gene bodies. Notably, the hurricane-associated differentially methylated regions identified in these macaques showed significant overlap with age-associated regions in humans [23]. Nevertheless, a subsequent study found no significant association between Hurricane Maria exposure and epigenetic clock acceleration in rhesus macaques when applying this dataset (*p* = 0.24) [39].

In a study by Sadoughi et al. (2026) [22], the authors investigated the effects of early-life adversity (ELA), including maternal loss, maternal primiparity, limited maternal kin network, low matrilineal rank, the presence of a closely related younger sibling, and being born into a large social group, on methylation patterns across various tissues in rhesus macaques. They found that while different ELAs influenced distinct CpG sites, the effect of each specific adversity was consistent across all tissues. Most ELAs did not lead to epigenetic clock acceleration, with the sole exception of maternal loss, which was associated with a +1.49-year acceleration in the pituitary gland. Notably, ELA-associated CpGs showed substantial overlap with age-associated ones; however, concordance in the direction of effect was observed only in the pituitary gland (Odds ratio = 3.3). In humans, stressors, particularly those experienced in early childhood, have been shown to significantly influence the rate of epigenetic aging and accelerate the epigenetic clock [47].

In summary, while epigenetic clocks in primates are a valuable and promising method for investigating the biology of aging and its relevance to humans, interpreting their results demands careful attention to numerous interacting elements (Figure 2C). Overall, the evidence highlights that epigenetic aging is not a fixed or predetermined process, but a dynamic biological result influenced by a complex combination of social, environmental, and metabolic factors. Grasping these influences is essential for accurately interpreting epigenetic clock data and for furthering their use in both primate studies and human aging research.

## 4. Aging-Associated Non-Coding RNA Markers and RNA Modifications

Among epigenetic factors, RNA molecules and their post-transcriptional modifications play a pivotal role, acting as both direct and indirect markers of age-related changes. Non-coding RNAs—such as microRNAs (miRNAs), long non-coding RNAs (lncRNAs), and circular RNAs (circRNAs)—function as direct epigenetic markers, as they actively participate in the regulation of gene expression and chromatin dynamics. Specifically, miRNAs regulate gene expression at the post-transcriptional level by binding to target mRNAs and repressing their translation or inducing their degradation; lncRNAs interact with chromatin, transcription factors, and other molecules, thereby influencing chromatin structure and gene activity, and circRNAs act as regulators of miRNAs, modulating their availability and participating in splicing and translation. Therefore, alterations in the expression and modification of non-coding profoundly affect gene expression profiles, with these changes becoming particularly pronounced during aging.

A recent multi-omics study of rhesus macaques analyzed 49 whole blood samples from individuals aged 2 to 34 years across four age groups. Pairwise comparisons identified 532 differentially expressed (DE) mRNAs, 250 DE lncRNAs, and 233 DE circRNAs. Notably, in the critical aging period of 11–19 years, 54.4% of detected lncRNAs showed increased expression. Genes coexpressed with these lncRNAs are involved in several cancer-related signaling pathways due to KEGG analysis [48]. The observed increase in lncRNA activity with age is also shown in the brains of rhesus macaques [49]. This process can alter gene expression, suggesting that lncRNAs play an important role in aging. Further analysis of abundant blood transcripts yielded 16 mRNAs, 4 lncRNAs, and 12 circRNAs with potential roles in aging. Eight of these, including lncRNA *XLOC_007571* and four circRNAs, *circ_0002743*, *circ_0005016*, *circ_0010527*, and *circ_0008814,* were also differentially expressed in the brain. These findings suggest a potential eight-biomarker panel for non-invasive assessment of brain aging, though large-scale human validation is needed before clinical application [48].

From an epigenetic perspective at the RNA level, a key regulator is the m6A modification of mRNA molecules, which directly influences transcript stability, localization, and translation. In a study by Zeming Wu and colleagues, m6A epitranscriptomes were characterized in the liver, cardiac muscle, and skeletal muscle of cynomolgus monkeys divided into two age groups: 4–6 years (young) and 18–21 years (old). Analysis of the relationship between RNA methylation dynamics and gene expression revealed that age-related changes in m6A predominantly affected mRNAs with high expression variability, consistent with tissue-specific aging signatures. Across tissues, a decline in m6A intensity was most pronounced in skeletal muscle. This reduction was explained by a 50% decrease in METTL3 protein levels in aging tissues, METTL3 being a key enzyme in m6A metabolism [50]. Functional experiments in human embryonic stem cells confirmed that METTL3 deficiency leads to decreased m6A levels and promotes accelerated cellular aging [51]. Collectively, these findings establish m6A modification of mRNA as a significant tissue-specific marker in primate epigenetic aging, with METTL3 levels serving as a potential indicator of these age-related epitranscriptomic changes.

**Figure 3:**
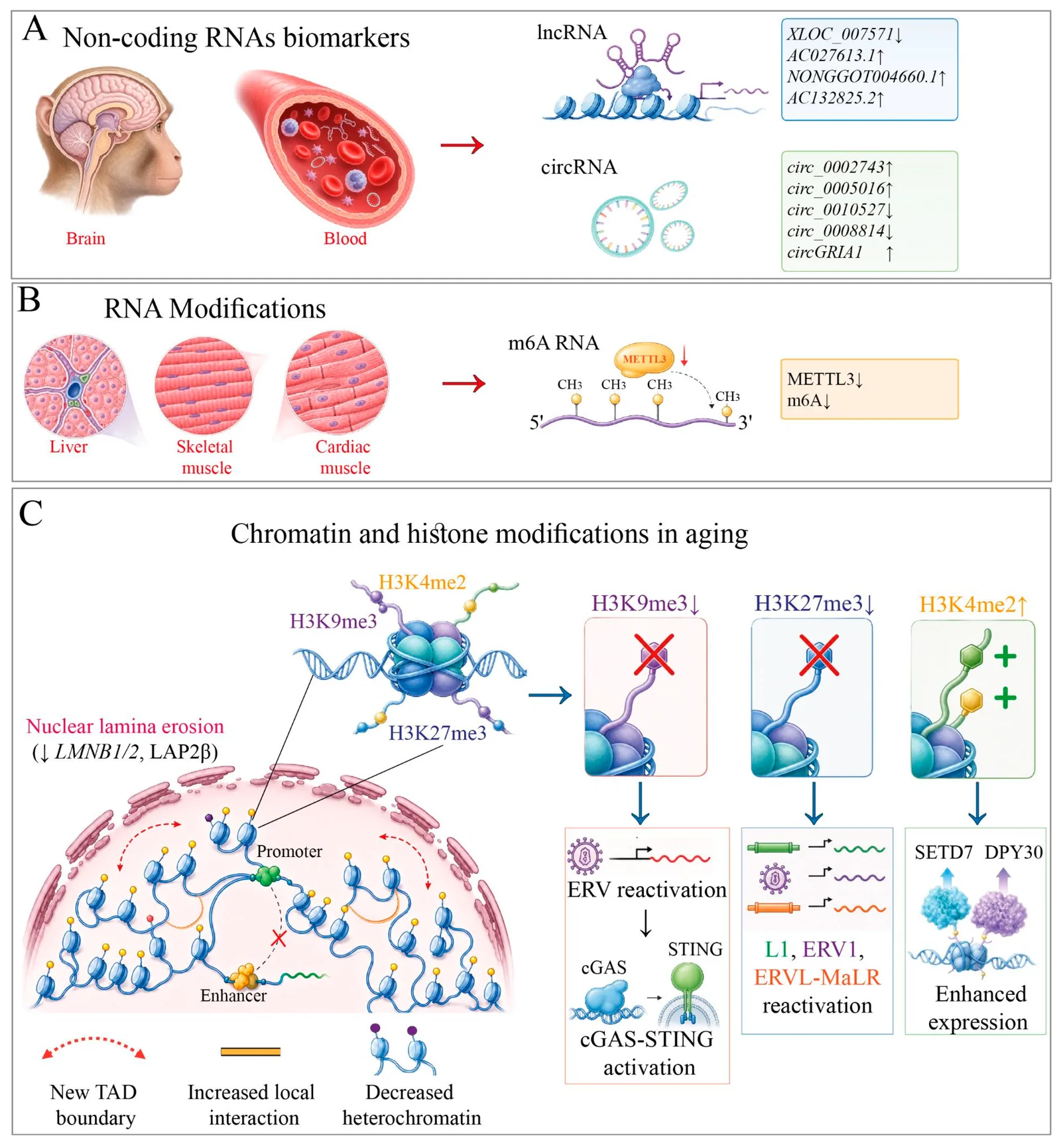
RNA and chromatin epigenetic mechanisms of aging in NHPs. (**A**) Non-coding RNA-based biomarkers in primate aging. (**B**) Age-related decline in m6A RNA modifications in NHP tissues. (**C**) Chromatin disorganization and histone modification alterations in primate aging. ↑—increase; ↓—decrease.

In conclusion, while the discussed non-coding RNAs serve as markers of epigenetic aging due to their age-dependent expression, their exact molecular targets remain elusive, warranting further investigation into their regulatory mechanisms in the aging process (Figure 3A). Importantly, studies in NHPs reveal that age-associated changes in non-coding RNA expression mirror systemic molecular alterations, with striking parallels observed across species, establishing NHPs as invaluable models for translating these findings to humans. Conversely, an age-dependent decline in the METTL3 enzyme acts as a functional modulator of aging, directly driving the reduction of m6A mRNA modifications across different tissue types (Figure 3B). Elucidating these regulatory mechanisms at the RNA level may ultimately pave the way for innovative diagnostic and therapeutic strategies to combat age-related decline.

## 5. Chromatin Organization and Histone Modifications

Post-translational modifications of histone termini, specifically H3K4me3 (a marker of active promoters), H3K27me3 (a mark of facultative heterochromatin), and H3K9me3 (associated with constitutive heterochromatin), represent major epigenetic determinants of chromatin state. Their age-dependent redistribution contributes to transcriptional noise, loss of cellular identity, and transposon reactivation, collectively constituting a prominent molecular signature of mammalian brain aging [52,53].

### 5.1. Age-Associated Histone Marks and Chromatin State Alterations

In NHPs, these marks are being investigated via ChIP-seq in the brains of rhesus macaques and baboons, providing organism-level evidence that complements and extends findings obtained in mice and humans. Using assays for open chromatin regions and higher-order chromatin architecture in the prefrontal cortex (PFC) of rhesus macaques, Ning et al. (2024) generated an epigenomic atlas across seven developmental stages, spanning both embryonic and postnatal periods, thereby covering all key phases of PFC maturation. This effort yielded 70 ChIP-seq and 14 Hi-C datasets. Comparative analysis between young (4.5 years) and aged (20 years) animals revealed several aging-related epigenetic alterations. These included a reduction in long-range chromatin interactions, leading to a loss of gene enhancer–promoter contacts, and an increase in local interactions, resulting in the disruption of topologically associating domains (TADs) and the formation of new TAD boundaries. Cross-species comparison with human PFC samples from 29- and 90-year-old individuals confirmed the evolutionary conservation of this aging-related shift in the balance of chromatin interactions. Loss of repressive H3K27me3 mark at the 20-year stage within various transposable elements (including L1, ERV1, and ERVL-MaLR) suggests that this epigenetic alteration drives their age-related activation [54].

In a complementary study, Zhang et al. (2023) investigated the molecular mechanisms of frontal lobe aging in cynomolgus macaques (*Macaca fascicularis*) aged 4–6 years (young) and 18–21 years (aged). They found that aging was associated with nuclear lamina erosion (accompanied by decreased expression of *LMNB1* and *LMNB2* and reduced LAP2β levels), loss of the heterochromatic mark H3K9me3, global DNA hypomethylation, and derepression of endogenous retroviruses (ERVs). Nuclear structure aberrations, leading to concomitant ERV reactivation, induced cGAS-STING-dependent neuroinflammation and neuronal senescence. These changes were recapitulated in vitro using human stem cell-derived neurons following knockdown of lamins B1 and B2. Importantly, pharmacological inhibition of ERV activity and cGAS-STING signaling partially ameliorated senescence markers. Moreover, treatment with the reverse transcriptase inhibitor abacavir attenuated aging phenotypes both in cellular models and in aged mice (18–30 months) [55]. These findings are consistent with the mechanistic framework proposed by Manning et al. (2025), in which H3K9me3 maintains chromocenter compaction and nuclear mechanical integrity; its loss is proposed to initiate a cascade of aging-related cellular deterioration [56]. Concurrently, a decrease in lamin B1 protein within the aging brain has been linked to neurodegenerative disorders, such as Alzheimer’s disease [57].

By contrast, Foret et al. (2014) employed ChIP-seq in neural progenitors of the subventricular zone in adult baboons and identified 863 genes enriched for H3K9me3. Notably, several targets involved in pathways orchestrating cell survival, apoptosis, proliferation, and stress responses, processes fundamentally intertwined with aging. Moreover, the H3K9me3 target set comprised 11 imprinted genes linked to central nervous system function and glial differentiation. The authors noted that H3K9me3 is critical for maintaining cell-type integrity in adult primate neurogenic niches, suggesting that its erosion with age could permit inappropriate expression of lineage-specific genes and drive age-related functional decline [58].

To investigate the postnatal and aging-related dynamics of the histone modification H3K4me2 in the PFC of rhesus macaques, Han et al. (2012) conducted a genome-wide ChIP-seq analysis across four age groups: 0.4, 9, 22, and 26 years. Their findings revealed a global increase in H3K4me2 occupancy at promoters and enhancers during both postnatal development and aging, accompanied by elevated expression of two H3K4 methyltransferases, SETD7 and DPY30, in the aging macaque brain. This increased expression likely underlies the progressive accumulation of this mark, thereby acting as a key driving factor of the aging process [59].

In summary, progressive chromatin disorganization and histone modification alterations emerge as a conserved hallmark of aging across NHPs and humans (Figure 3C).

### 5.2. Chromatin Deregulation and Histone Modification Shifts in Aging-Regulatory Genes

Crucially, the structural changes in chromatin conformation are tightly linked to the aberrant expression of critical aging-regulatory genes. Beyond general chromatin disorganization, Ning et al. (2024) analyzed the expression profiles of genes located within newly formed TAD boundaries in 20-year-old rhesus macaques. Among these, *HIF1A*, a key player in the oxidative stress response, was particularly prominent. Due to increased local chromatin interactions, the contacts between *HIF1A* and its cis-regulatory elements were significantly enhanced, driving its upregulation. The authors noted that such elevated expression of this gene in microglia has been previously associated with age-related neuroinflammatory processes. Concurrently, genes experiencing a loss of enhancer–promoter (E–P) contacts as a result of reduced long-range chromatin interactions showed a significant enrichment in gene sets associated with schizophrenia, dementia, and Alzheimer’s disease. Thus, the emergence of novel chromatin contacts or the disruption of existing ones may serve as a critical factor triggering the development of age-related neurodegeneration [54].

Although the majority of the data reflect correlative shifts in histone marks during aging, specific enzymes, such as SETD7 and DPY30, have been identified that likely play a causal role in the age-dependent accumulation of the H3K4me2 modification [59].

Nevertheless, functional studies aimed at providing a comprehensive understanding of how chromatin reorganization and histone modifications mechanically drive downstream aging pathways remain limited.

## 6. Sexual Dimorphism in Aging-Associated Epigenetics in NHPs

Human longevity represents a sexually dimorphic trait [60], and comparable patterns of sexual dimorphism have been documented across primate taxa, prompting several studies to focus on sex-dependent aging-associated markers. In a comprehensive investigation by Horvath et al. (2023), DNA methylation profiles were examined in 2400 tissue samples derived from 37 primate species. The authors established that the majority of age-related DNA methylation changes are tissue-specific in nature and delineated a suite of CpG sites consistently associated with sex across multiple primate species and tissue types. Although most of these sex-associated markers are localized to the X chromosome, consistent with X-chromosome inactivation in females, minimal overlap was observed between CpGs linked to age and those linked to sex. On this basis, the authors concluded that epigenetic alterations driven by aging and those attributable to sex represent largely distinct biological processes [20]. A similar pattern was observed in marmosets, which nevertheless represent a notable exception. While sex can generally be predicted with high accuracy from DNA methylation profiles in primates, much as in humans, marmosets stand out as a remarkable outlier. In this species, sex assignment accuracy based on methylation data was only 66%, barely above random (50%) and substantially lower than that reported for other mammals, where such models typically reach near 100% accuracy. When directly comparing males and females, not a single CpG site reached genome-wide significance. Even at a highly permissive threshold (alpha = 0.005), only a modest number of CpGs (62 hypermethylated and 12 hypomethylated in females) showed any detectable sex effect in marmoset blood samples. Consequently, due to this near-absence of sexual dimorphism in the methylome, aging-associated markers in these animals do not exhibit a sexually dimorphic trait. Collectively, these findings indicate that sexual dimorphism in the marmoset DNA methylome is strikingly weak, particularly when contrasted with the pronounced sex-associated methylation differences observed in all other mammalian species studied to date [25].

**Figure 4:**
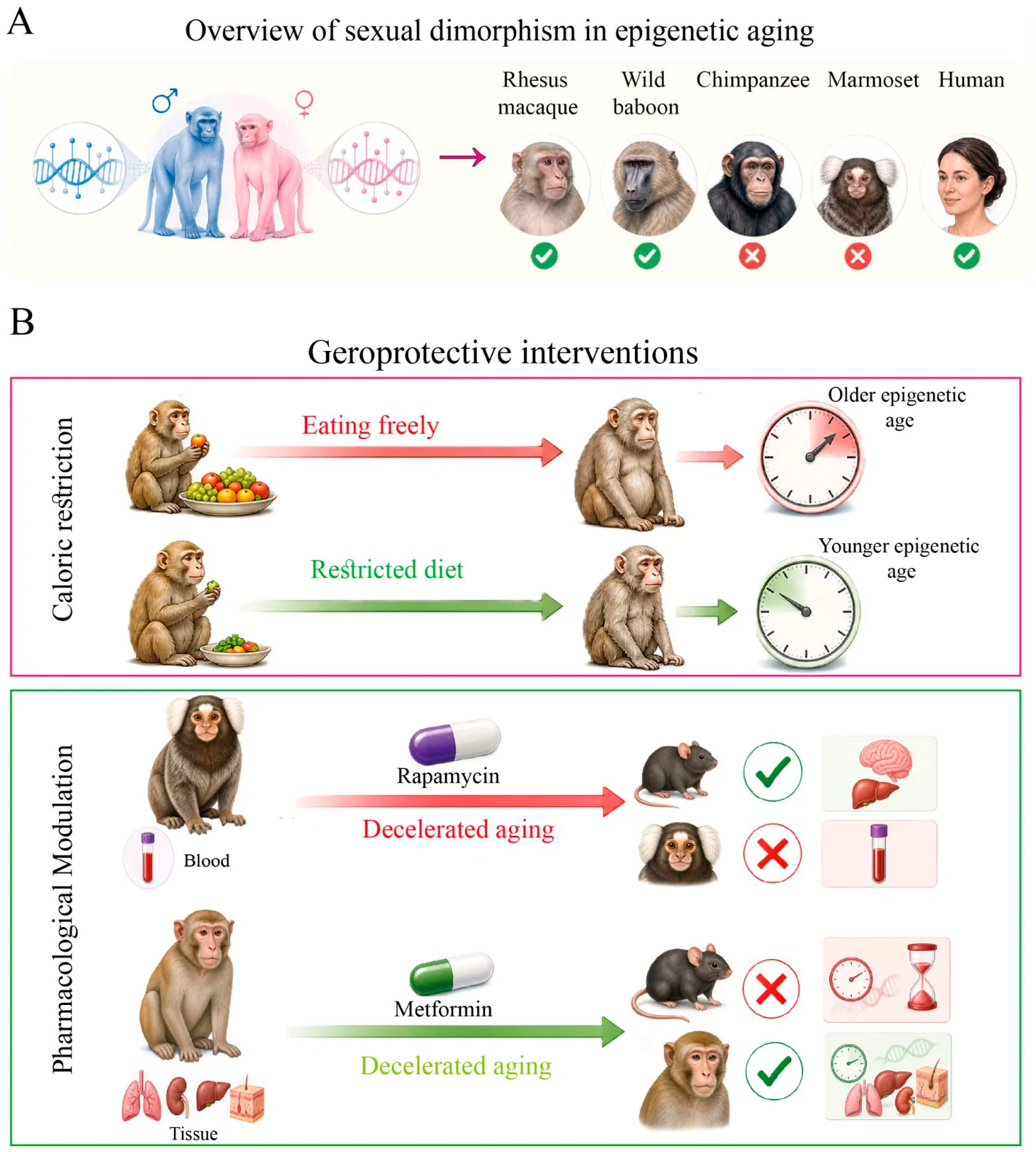
Intrinsic and extrinsic modulators of epigenetic aging in non-human primates. (**A**) Sexual dimorphism in epigenetic aging across NHP species. (**B**) Caloric restriction and pharmacological modulation of epigenetic aging.

However, the conclusion about low sexual dimorphism in aging-associated markers was challenged by studies employing rhesus macaques as a model organism, which demonstrate that the majority of age-associated DNA methylation alterations are sex-specific, with only minimal overlap between males and females. Specifically, analysis of DNA methylation patterns in the hippocampus and liver revealed that only 3% and 21% of age-associated methylation sites, respectively, are shared between the sexes. In both tissues, the number of differentially methylated sites associated with aging was greater in females than in males. Furthermore, the predominant direction of change was hypomethylation, with the vast majority of age-associated sites exhibiting a loss of methylation over time. Collectively, these findings provide compelling molecular evidence that the epigenetic trajectory of aging is fundamentally distinct between the sexes [61]. Supporting these data, application of a DNA methylation-based epigenetic clock to a wild baboon population in Kenya also reveals a marked sex-dependent relationship with chronological age. This sexual dimorphism is most clearly reflected in the regression slope between predicted epigenetic age and actual age, with males demonstrating a rate of epigenetic age acceleration 2.2 times higher than that of females per unit of chronological time. Importantly, this sex difference does not emerge among individuals younger than eight years, a developmental milestone that roughly corresponds to the life-history phase when most males attain dominant social standing and disperse from their natal groups. Instead, sexually divergent epigenetic aging trajectories become apparent only after individuals have reached full physiological maturity and social adulthood. This period coincides with the greatest divergence in male and female life-history strategies, a stage during which sex-specific rates of senescence are expected to become most pronounced [41]. An intriguing observation emerged regarding chimpanzees, our closest non-human primate relatives: no sex-based differences were detected in the rate of the epigenetic clock, whether measured using the chimpanzee-specific model or the classic Horvath clock [38]. In contrast, among humans, sex disparities in epigenetic aging are consistently observed across racial and ethnic groups, with males exhibiting a faster rate of aging [62] (Figure 4A).

Consistent with the sex-dependent patterns described above, Xu et al. (2020) reached a comparable conclusion through their investigation of molecular markers of brain aging in macaques. Specifically, they identified circGRIA1, a circular RNA derived from the *GRIA1* gene (which encodes the AMPA receptor subunit GluR1), as a prominent age-related transcriptional feature, further underscoring the sexually dimorphic nature of aging processes across primate species. Mechanistically, the downregulation of GluR1 was shown to contribute to age-dependent synaptic dysfunction, manifesting as reduced synaptogenesis, impaired synaptic plasticity, and disrupted calcium homeostasis. Notably, the age-associated upregulation of *circGRIA1* was observed exclusively in male macaques. Complementary in vitro experiments further demonstrated that circGRIA1 knockdown rescued synaptic and plasticity deficits solely in male-derived neurons, with no analogous effect in female cells. On the basis of these findings, the authors posit that circGRIA1 constitutes a male-specific mediator of age-related synaptic decline in the primate brain [63]. In a complementary study, comprehensive RNA-seq and CAGE-seq analyses were employed to characterize age-dependent dynamic changes in lncRNA expression in the rhesus macaque brain. Sex-specific expression patterns of both lncRNAs and mRNAs were observed across different ages. Specifically, three sex-biased lncRNAs: *AC027613.1*, *NONGGOT004660.1*, and *AC132825.2* were identified as abundantly expressed in the macaque brain and were found to correlate with age-specific characteristics [49].

Despite comparable global cellular composition, the majority of genes in the primate brain are expressed in a sex-dependent manner [64]. These findings collectively argue that sexual dimorphism is not a secondary but a central molecular feature particularly of brain aging, with implications for sex-specific disease vulnerability.

## 7. Calorie Restriction and Pharmacological Modulation of Epigenetic Aging

Given their evolutionary proximity to humans, NHPs represent the gold-standard model for late-stage preclinical evaluation of pharmacological interventions and longevity therapeutics, particularly when human clinical trials are ethically precluded. In this analysis, we contextualize NHP findings alongside rodent studies, with a focus on epigenetic outcomes, to assess the cross-species translatability of various geroprotective agents.

Caloric restriction remains one of the most extensively researched and efficacious geroprotective strategies. Its beneficial effects, including the activation of autophagy and the reduction of oxidative stress and systemic inflammation, are largely attributed to the downregulation of the insulin/IGF signaling pathway. This modulation is evident not only in metabolic processes but also at the epigenetic level, where it decelerates age-associated alterations in DNA methylation [65,66]. In a study of rhesus macaques aged 22–30 years, individuals subjected to a 30% calorie-restricted diet from adulthood (7–14 years) through old age exhibited whole-blood methylation indices indicating a biological age approximately 7 years younger than their chronological age, relative to ad libitum-fed controls. In contrast, macaques with elevated body mass index (obesity) displayed accelerated epigenetic aging in whole blood [14]. Similarly, in mice, early-life calorie restriction initiated at 14 weeks of age robustly and significantly reduced epigenetic age in cohorts aged 10, 18, 23, and 27 months, with the effect being most pronounced in older age groups [67].

Among geroprotective agents investigated in non-human primates, rapamycin, an inhibitor of the mTOR signaling pathway, has attracted considerable interest due to its ability to stimulate autophagy and suppress age-related chronic inflammation [68]. In a 2021 study, Horvath et al. evaluated the effects of rapamycin on DNA methylation profiles and epigenetic clock speed in common marmosets (aged 5.66–13.4 years) treated for 2–3.5 years. The drug did not decelerate epigenetic aging in blood. Although rapamycin induced average hypermethylation across genomic regions, no statistically significant epigenetic effect was observed; at decreasing significance thresholds, only 48 CpG sites were altered. Notable among these were exons of *HECW2* (hypermethylated) and *EMC7* (hypomethylated), as well as the *SP1* transcription factor motif, a key regulator of the mTORC1/P70S6K/S6 signaling pathway and a factor associated with age-related pathologies including hypertension, atherosclerosis, and Alzheimer’s disease [25]. In mice, rapamycin strongly slows epigenetic aging in liver and brain tissue, suggesting a tissue-specific effect [69,70]. Despite this limitation, rapamycin has been shown to extend lifespan and delay age-related pathologies in rodent models [71,72]. Notably, unlike in NHPs, long-term rapamycin administration in mice is associated with adverse metabolic effects, including hyperlipidemia and hyperglycemia [73].

Another well-characterized geroprotective agent is metformin, which operates through multiple interconnected mechanisms: activation of AMPK, inhibition of insulin/IGF-1 and mTORC1 signaling, stimulation of autophagy, suppression of inflammation, and reduction of oxidative stress [74,75]. In a study of cynomolgus macaques (aged 13–16 years), long-term metformin administration induced systemic slowing of molecular aging, as evidenced by changes in gene expression, DNA methylation, chromatin organization, and proteomic and metabolomic profiles. Notably, deceleration of epigenetic aging, measured by the DNAmAge clock, was observed across multiple tissues, including lung, kidney, liver, and skin. Furthermore, metformin effectively restored H3K9me3 heterochromatin levels, a modification associated with suppression of endogenous retroviruses, whose expression increases with age [76]. In contrast, findings in conventional mouse models have been less consistent. A systematic meta-analysis of mouse studies reported no statistically significant lifespan extension with metformin, revealing instead both dose- and time-dependent effects [77].

Overall, the data demonstrate that while CR exhibits conserved epigenetic benefits across species, pharmacological interventions display variable translatability (Figure 4B). However, despite the irreplaceable physiological relevance of NHPs for specific endpoints, their use is increasingly constrained by mounting regulatory pressures. The U.S. Food and Drug Administration, in particular, has championed a paradigm shift toward alternative platforms, including microphysiological organ-on-a-chip systems, in silico toxicology, and advanced computational risk modeling [78].

## 8. Perspectives and Conclusions

In this study, we identified markers of aging-associated epigenetic changes in NHPs across multiple levels of regulation. A comparative review of the literature further revealed that several of these markers are conserved in NHPs and also correlate with human aging (Table 2). These findings underscore the unique value of NHPs, particularly great apes, for investigating the evolutionary conservation of epigenetic aging mechanisms. Nevertheless, the use of great apes is severely constrained by ethical and practical challenges. Their advanced cognitive abilities, extended childhoods, and complex social structures demand specialized housing and care, and largely preclude invasive experimental approaches. Consequently, macaques and baboons have emerged as the most pragmatic and widely adopted models, offering a viable compromise between phylogenetic proximity to humans and experimental tractability.

The study of age-related epigenetic changes between humans and NHPs remains a largely unexplored territory. While the development of the HorvathMammalMethylChip40 microarray has facilitated rapid cross-species comparisons of major age-associated epigenetic alterations, the choice of analytical platform profoundly influences research findings. Although widely applied, this array is limited to CpG sites that are conserved across mammals, potentially missing lineage-specific epigenetic dynamics. By contrast, genome-wide methodologies such as RRBS offer more extensive coverage and the capacity to uncover unique species-specific methylation signatures, rendering them more appropriate for exploratory and comparative investigations.

The interpretation of epigenetic data is further complicated by tissue selection. Blood, due to its ease of collection, continues to be the primary tissue for constructing epigenetic clocks. Nevertheless, studies incorporating multiple tissues consistently demonstrate that the majority of age-related methylation changes are tissue-specific, with only a small subset of markers being universally conserved. Furthermore, the pace of epigenetic aging varies across tissues, for instance, chimpanzee brain tissue shows slower age-related methylation changes than blood [43]. Such heterogeneity highlights the necessity of advancing beyond bulk-tissue analyses to single-cell resolution. Recent work in mice has demonstrated that cell-type-specific clocks can capture the distinct aging rates of individual cell populations, such as neurons and astrocytes, while minimizing confounding effects from age-related shifts in cellular composition [111,112]. Translating these approaches to primates is now a key priority in epigenomic research.

Notwithstanding these developments, considerable gaps persist. Among the great apes, species-specific epigenetic clocks are available only for chimpanzees [26,38]; orangutans and gorillas are still limited to a universal pan-primate age predictor [20]. Gibbons pose an even greater challenge, as their genomes are characterized by extensive chromosomal rearrangements and abundant retrotransposons, which complicate methylome data analysis [113,114]. The lack of tailored clocks for the majority of primate species obstructs efforts to distinguish between inherited and environmentally influenced methylation patterns, a crucial distinction for comprehending the evolutionary biology of aging. In response to these challenges, non-invasive approaches have garnered increasing attention. One particularly promising direction is the microbiome clock, which, in baboons, has been shown to accurately estimate chronological age based on fecal microbial composition, thereby bypassing the ethical and logistical difficulties associated with tissue sampling [115]. Ultimately, the most comprehensive and robust insights into primate epigenetic aging will emerge from integrative strategies that combine genome-wide methylation profiling, multi-tissue and single-cell analyses, and innovative non-invasive biomarkers. Such an integrated framework will not only reveal species- and tissue-specific aging signatures but also deepen our understanding of both the conserved and divergent features of the aging process across primates.

In summary, the reviewed evidence indicates that NHPs occupy a distinctive position as models for studying epigenetic aging, providing causal insights that are ethically and practically unfeasible in human research. However, the predominance of tissue-specific and lineage-specific epigenetic modifications necessitates caution against broad generalizations derived from a single tissue type or species. The principal value of NHP models resides not in their capacity to exactly replicate human aging processes, but rather in their ability to identify epigenetic mechanisms that are evolutionarily conserved and thus more likely to have translational relevance, as opposed to those representing species- or tissue-specific adaptations. This differentiation is essential for effectively prioritizing targets for geroprotective therapeutic interventions.

## Figures and Tables

**Table 1:** Epigenetic clocks in NHPs.

Species	Method	Clock Type	Tissue	Age Range and Sex	Individuals/Samples	Performance	Presence of Human Tissues	Reference
Chimpanzees	Pyrosequencing (14 CpG from: *ELOVL2 (9)*, *CCDC102B (2)*, and *ZNF423 (3)*)	*ELOVL2* (*CpG-7,3,1,6*) + *CCDC102B* (*CpG-1*)	Blood	2–39 years (sex NR)	20 individuals	R = 0.741, MAD**^1^** = 5.42 years	No	[26]
Chimpanzees	Illumina Infinium Methylation EPIC array	Chimpanzee-specific (80 CpG sites)	Blood	1–58 years (sex NR)	83 individuals/113 samples	MAD**^2^** = 2.4 years	No	[38]
Rhesus macaques	HorvathMammalMethylChip40	Macaque-specific: pan-tissue, blood, skin; Human–macaque: chron. age, relative age	Blood (n = 199), skin (n = 51), other tissue (n < 7)	1.8–42 years (blood: 71 F, 128 M; skin: 13 F, 38 M)	281 individuals	Macaque pan-tissue: R = 0.95, MAE = 1.4 years; Human–macaque chron. age: R = 0.98, relative age: R ≥ 0.97 (blood and skin)	Human–macaque clocks	[21]
Rhesus macaques (free-ranging)	RRBS	Site-based, window-based	Blood	1.44 months–28.82 years (273 F, 220 M)	493 individuals/563 samples	Site-based: R = 0.82, MAD**^2^** = 2.11 years; Window-based: R = 0.9, MAD**^2^** = 1.42 years	No	[39]
Rhesus macaques (semi-free ranging)	RRBS	Tissue-specific	14 tissues	1.5 months–25.9 years (132 F, 105 M)	237 individuals/2485 samples	R = 0.81 (ovary)—0.95 (liver), MAE = 0.82 (adipose)—1.53 (ovary)	No	[22]
Baboons (wild)	RRBS	Baboon-specific (573 CpG sites)	Blood	48 M, 22 F [40] + 142 F, 135 M; Avg. lifespan: 10.3 years (F), 7.9 years (M)	70 + 245 individuals/277 samples	MAD**^3^** = 1.1 years, R = 0.762	No	[41]
Olive–yellow baboon hybrids	HorvathMammalMethylChip40	Baboon-specific: pan-tissue, tissue-specific; Human–baboon: chron. age, relative age	Cortex (n = 105), heart (n = 48), adipose (n = 41), cerebellum (n = 38), liver (n = 50), muscle (n = 44)	Late fetal—22.8 years	326 samples	Baboon pan-tissue: R = 0.96, MAE = 1.1 years; Human–baboon chron. age: R = 0.99, relative age: R = 0.97	Human–baboon clocks	[20]
Olive baboons (captive)	RRBS	Baboon-specific (153 CpG sites)	Blood	1.1–19.33 years (118 F, 22 M)	140 individuals	NR	No	[42]
Pan-primate (37 species)	HorvathMammalMethylChip40	Chron. age, relative age	Multiple (tissue-dependent)	Species-dependent	2398 tissue samples	Chron. age: R = 0.99, relative age: R = 0.96	Universal pan-primate clock	[20]
Vervet monkeys	HorvathMammalMethylChip40	Vervet-specific: multi-tissue, blood, liver, brain cortex; Human–vervet: chron. age, relative age	Blood (n = 144), liver (n = 48), cortex BA10 (n = 48)	Blood: 0–25 years, liver: 0–21 years, BA10: 0–22 years	240 samples	Vervet multi-tissue: R = 0.98, MAE = 0.89 years; Human–vervet chron. age: R = 0.99, relative age: R = 0.98	Human–vervet clocks	[24]
Common marmosets	HorvathMammalMethylChip40	Marmoset-specific pan-tissue (trained on blood); Human–marmoset: chron. age, relative age	Blood	0.5–15.5 years (28 F, 30 M)	58 samples	Marmoset pan-tissue: R = 0.95, MAE = 0.72 years; Human–marmoset relative age: R = 0.96	Human–marmoset clocks	[25]

F—female; M—male; Avg.—average; NR—not reported; RRBS—Reduced Representation Bisulfite Sequencing; chron.—chronological; MAE—median absolute error; MAD**^1^**—mean absolute deviation; MAD**^2^**—median absolute deviation; MAD**^3^**—median absolute difference; R—correlation coefficient; BA10—Brodmann area 10. For studies that provided both pan-tissue and tissue-specific models, only the performance metrics for the pan-tissue or multi-tissue versions are presented, as these are considered most representative for cross-tissue comparative analyses.

**Table 2:** Epigenetic markers of aging in NHPs.

Epigenetic Mechanism		Age-Related Marker in HNPs	Association with Aging or Age-Related Pathologies in Humans	Reference
DNA	hypermethylation	PRC2 targets & binding sites	Yes	[19,20,24,79]
		*KLF14*	Yes	[19,20,80]
		ZBTB family transcription factors	Yes	[23,81]
		*NRF1*	Yes	[23,82]
		*LHFPL4, LHFPL3*	Yes	[19,20,24]
		*FOXD3*	Yes	[20,24]
		*TLX3*	Yes	[19,24]
		*ANK1*	Yes	[25,83]
		*SCG3*	Yes	[25,84]
		*UNC79*	ND	[25]
		*ELOVL2*	Yes	[26,27]
		*HOX* genes	Yes	[20,79,85,86]
		*BDNF*	Yes	[20,87,88]
		*TBR1*	Yes	[20,89]
		*FOXG1*	Yes	[20,90]
	hypomethylation	*LARP1*	Yes	[19,91]
		*Bach1*	Yes	[23,92,93]
		*NFE2L2*	Yes	[23,94,95]
		*JUND*	Yes	[23,96]
		*JUN*	Yes	[23,96]
		*MAFK*	Yes	[23,96]
		*FOS*	Yes	[23,96]
		*FOSL2*	Yes	[23,96,97]
		*SP1*	Yes	[24,98]
		*CD46*	Yes	[24,99]
		*TRPS1*	Yes	[20,24]
		*SNX1*	Yes	[20,100]
		*SMG6*	Yes	[20,101]
		*ARID5B*	Yes	[20,102]
		*EWSR1*	Yes	[20,103]
		TEs	Yes	[23,30]
RNA	circRNA	*circ_0002743* ↑	ND	[48]
		*circ_0005016* ↑	ND	[48]
		*circ_0010527* ↓	ND	[48]
		*circ_0008814* ↓	ND	[48]
		*circGRIA1* ↑	Yes	[63,104]
	lncRNA	*XLOC_007571* ↓	ND	[48]
		*AC027613.1* ↑	ND	[49]
		*NONGGOT004660.1* ↑	ND	[49]
		*AC132825.2* ↑	ND	[49]
	m^6^A RNA	METTL3 ↓→m^6^A ↓	Yes	[50,51]
Histone modification	H3K27me3 ↓	L1 ↑	Yes	[54,105]
		ERV1 ↑	Yes	[54,106]
		ERVL-MaLR ↑	Yes	[54,107]
	H3K9me3 ↓	ERV ↑→cGAS-STING ↑	Yes	[55,106,108]
	H3K4me2 ↑	SETD7 ↑→H3K4me2 ↑	Yes	[59,109]
		DPY30 ↑→H3K4me2 ↑	Yes	[59,110]
Nuclear lamina erosion		ERV ↑→cGAS-STING ↑	Yes	[55,106,108]
Chromatin interactions		Local ↑→split of TADs	Yes	[54]
		Distant ↓→loss of E–P contacts	Yes	[54]

TEs—transposable elements; ERV—endogenous retrovirus; TADs—topologically associating domains; ↑—increase; ↓—decrease; E–P—enhancer–promoter; Yes—direct association with human aging reported; ND (not determined)—not yet tested in humans.

## Data Availability

No new data were created or analyzed in this study. Data sharing is not applicable to this article.

## Abbreviations

The following abbreviations are used in this manuscript:

NHPs	Non-human primates
TEs	Transposable elements
PRC2	Polycomb repressive complex 2
RRBS	Reduced Representation Bisulfite Sequencing
R	Correlation coefficient
MAE	median absolute error
MAD**^1^**	mean absolute deviation
MAD**^2^**	median absolute deviation
MAD**^3^**	median absolute difference
ELA	early-life adversity
ncRNA	non-coding RNA
miRNAs	microRNAs
lncRNAs	long non-coding RNAs
circRNAs	circular RNAs
DE	differentially expressed
PFC	prefrontal cortex
TAD	topologically associating domain
ERVs	endogenous retroviruses
E-P	enhancer–promoter
